# Early prediction of histopathological response of rectal tumors after one week of preoperative radiochemotherapy using ^18^ F-FDG PET-CT imaging. A prospective clinical study

**DOI:** 10.1186/1748-717X-7-124

**Published:** 2012-08-01

**Authors:** Natalia Goldberg, Yulia Kundel, Ofer Purim, Hanna Bernstine, Noa Gordon, Sara Morgenstern, Efraim Idelevich, Nir Wasserberg, Aaron Sulkes, David Groshar, Baruch Brenner

**Affiliations:** 1 Department of Radiology, Rabin Medical Center, Beilinson Hospital, Petah Tiqva, Israel; 2 Institute of Oncology, Davidoff Center, Rabin Medical Center, Beilinson Hospital, Petah Tiqva, Israel; 3 Department of Nuclear Medicine, Rabin Medical Center, Beilinson Hospital, Petah Tiqva, Israel; 4 Department of Pathology, Rabin Medical Center, Beilinson Hospital, Petah Tiqva, Israel; 5 Department of Surgery B, Rabin Medical Center, Beilinson Hospital, Petah Tiqva, Israel; 6 Sackler Faculty of Medicine, Tel Aviv University, Tel Aviv, Israel; 7 Institute of Oncology, Kaplan Medical Center, Rehovot, Israel; 8 Department of Nuclear Medicine, Assuta Medical Center, Tel Aviv, Israel

**Keywords:** Radiochemotherapy, Rectal cancer, PET-CT

## Abstract

**Background:**

Preoperative radiochemotherapy (RCT) is standard in locally advanced rectal cancer (LARC). Initial data suggest that the tumor’s metabolic response, i.e. reduction of its ^18^ F-FDG uptake compared with the baseline, observed after two weeks of RCT, may correlate with histopathological response. This prospective study evaluated the ability of a very early metabolic response, seen after only one week of RCT, to predict the histopathological response to treatment.

**Methods:**

Twenty patients with LARC who received standard RCT regimen followed by radical surgery participated in this study. Maximum standardized uptake value (SUV-MAX), measured by PET-CT imaging at baseline and on day 8 of RCT, and the changes in FDG uptake (ΔSUV-MAX), were compared with the histopathological response at surgery. Response was classified by tumor regression grade (TRG) and by achievement of pathological complete response (pCR).

**Results:**

Absolute SUV-MAX values at both time points did not correlate with histopathological response. However, patients with pCR had a larger drop in SUV-MAX after one week of RCT (median: -35.31% vs −18.42%, p = 0.046). In contrast, TRG did not correlate with ΔSUV-MAX. The changes in FGD-uptake predicted accurately the achievement of pCR: only patients with a decrease of more than 32% in SUV-MAX had pCR while none of those whose tumors did not show any decrease in SUV-MAX had pCR.

**Conclusions:**

A decrease in ΔSUV-MAX after only one week of RCT for LARC may be able to predict the achievement of pCR in the post-RCT surgical specimen. Validation in a larger independent cohort is planned.

## Background

Preoperative radiochemotherapy (RCT) is standard in locally advanced rectal cancer (LARC) [1-3]. This treatment is highly effective; tumor down-staging is common and in 15-30% of patients a pathological complete response (pCR) is achieved [4,5]. In light of the correlation between tumor histopathological response and patient outcome, this response is commonly used as a surrogate for efficacy [6-8]. However, histopathological response is known only after surgery, when RCT can no longer be modified. Therefore, attempts are being made to develop methods for its early prediction.

One of the leading candidate predictive markers for histopathological response is 18-fluorodeoxyglucose (FDG) positron emission tomography (PET)-computerized tomography (CT). Multiple studies reported a correlation between metabolic response, i.e. the reduction of the tumor’s FDG uptake following RCT, and histopathological response in LARC [5,9-13]. However, most studies evaluated the metabolic response by comparing the baseline PET-CT scan, done just before RCT, and a scan done after its completion [5,9-12]. Yet, such information does not enable modification or interruption of RCT. Few studies reported an earlier prediction of histopathological response in LARC based on PET-CT performed during RCT [13-19]. Nevertheless, data on prediction of response to preoperative therapy for LARC using early metabolic response as a surrogate are still very limited, and are almost invariably based on PET-CT done toward the midst of RCT. The purpose of our study was to prospectively evaluate the ability of a very early metabolic response, after only one week of RCT, to predict response to treatment.

## Methods

### Patients

This prospective study included 20 patients who received preoperative RCT for LARC at the Rabin Medical Center (RMC), Beilinson Hospital, Israel, between February 2008 and July 2009. The study was approved by the institutional ethics committee and written informed consent was obtained. Eligibility criteria included histologically confirmed primary (non-recurrent) LARC, defined as uT3-4NxM0 or uTxN + M0 disease according to the American Joint Committee on Cancer (AJCC) version 7 [20]. Baseline staging work-up included rigid proctorectoscopy, endoscopic ultrasound (EUS) and whole body PET-CT. Other inclusion criteria included FDG-avid tumors, Eastern Cooperative Oncology Group (ECOG) performance status <2 and good organ function. All patients were planned for preoperative RCT followed by surgery. The main exclusion criteria included prior chemotherapy or radiotherapy for any cause or surgery for rectal cancer and hypersensitivity to ^18^FDG.

### Radiochemotherapy

RCT consisted of radiotherapy (RT) with concurrent fluoropyrimidine-based chemotherapy. The RT protocol was standard: 45 Gray (Gy) delivered in 1.8 Gy daily fractions, 5 times per week, and a boost to the tumor of 5.4-9.0 Gy. The dose was prescribed to the isodose encompassing the primary tumor and the internal iliac nodes using 6 or 18 MV photons.

Chemotherapy started on the first day of RT and continued until its completion. It consisted of one of three regimens: continuous infusion (CI) of 5-fluorouracil (5FU) 180 mg/m^2^/d or oral administration of capecitabine 825 mg/m^2^ x 2/d or UFT 300 mg/m^2^/d, all given on the days of RT delivery.

### PET-CT evaluation protocol

Patients performed a baseline PET-CT within two weeks prior to initiation of RCT. A second scan was done after one week of treatment, with 24 hour confidence margins (i.e. scans were done after 6–8 days of RCT).

### Surgery

Prior to surgery, patients were restaged with rigid proctorectoscopy, abdominal and pelvic CT, EUS and occasionally PET-CT. Radical surgery, including total mesorectal excision (TME), was scheduled 6–10 weeks after the completion of RCT. The type of surgery, low anterior resection (LAR) or abdominoperineal resection (APR), was at the discretion of the surgeon.

### Histopathological tumor response evaluation

Histopathological tumor response was determined by a single expert pathologist (SM), who was blinded to the corresponding metabolic response. Response was classified twice, by achievement of pCR and by tumor regression grade (TRG). TRG was classified as proposed by Mandard et al. [21], from complete tumor response (TRG I) gradually to no regressive changes within the tumor (TRG V). As data regarding the separation of responders and non-responders according to the TRG classification are inconsistent [7,22,23], patients were compared using individual as well as combined categories (e.g. I-II vs III-V). While TRG relates only to the primary tumor, pCR relates to the nodal status too. Accordingly, pCR was defined as no evidence of residual tumor, neither in the rectal wall (pT0) nor in the regional lymph nodes (pN0).

### PET-CT imaging and processing

Patients fasted for a minimum of 4 hours and blood sugar level was confirmed to be lower than 200 mg/dl immediately before the intravenous injection of 370–666 MBq of FDG. All images were obtained 60 minutes later by using an integrated eight-sectiony PET-CT scanner (Discovery ST; GE Medical Systems, Milwaukee, Wis).

Patients drank oral contrast fluid (300 mg Telebrix). Parameters for limited CT scan of the region of interest (pelvis) were identical for both studies as follows: helical CT at 0.8 second/rotation; 100–300 mAs; 120 kVp; section thickness, 3.75 mm with 3.75-mm interval. Iodine contrast medium (Ultravist 300) was administered intravenously during CT scan, unless iodine allergy, borderline renal function or patient refusal. Immediately after CT, PET was performed. The acquisition time for emission scans was 3–4 minutes per bed position with a one-section overlap CT data used for attenuation correction. Images were reconstructed with a standard iterative algorithm.

### PET-CT analysis

Visual and semi-quantitative analysis was performed and the maximum standardized uptake value (SUV-MAX) calculated in the rectal lesion at baseline and after one week of RCT. The changes were expressed as the percentage of SUV reduction (ΔSUV-MAX). Each PET-CT reading was done separately by two expert radiologists (NG, HB), who were blinded to the timing of the study and to any information on clinical or histological response. The final results were averages of the two separate readings.

### Statistical analysis

To evaluate the separation between patients who achieved pCR and those who did not and between responders and non-responders per TRG regarding the change of SUV-MAX values between baseline and day 8 PET-CTs, the Mann–Whitney test was used. As response per TRG is still unsettled (see above), the change of SUV-MAX values were also compared between individual TRG categories using the Kruskal-Wallis test. Receiver operating characteristic (ROC) analysis was used to find a cutoff for the tests. Analyses were done two-sided at a 5% significance level.

To define the study’s sample size, we assumed that a 15% decrease in tumor SUV-MAX value will identify metabolic response, based on the recommendations of the European Organization for Research and Treatment of Cancer (EORTC) PET study group [24]. Hence, the minimal sample size required to achieve a 95% power level was 16 patients. Our sample size was therefore 20 patients.

## Results

### Patients

Twenty patients were included in the study. The patient and tumor characteristics at presentation are summarized in Table 1. The median age was 65 years, with an even gender distribution. The majority of tumors (80%) were located ≥5 cm from the anus. There was a similar proportion of clinical stage II and III disease. When these data were available, most tumors were found to be well to moderately differentiated.

**Table 1:** Patient and tumor characteristic at presentation

	**Number**	**%**
Age, yrs		
Median (range)	65 (45–86)	
Gender		
Male	10	50
Female	10	50
Distance from anal verge		
< 5 cm	4	20
5-8 cm	11	55
>8 cm	5	25
Clinical T stage		
uT2	1	5
uT3	19	95
Clinical N stage		
uN0	16	80
uN+	4	20
Clinical TNM stage		
IIA (T3N0)	11	55
IIIB (T3-T4N1)	8	40
IIIC (TanyN2)	1	5
Grade		
I-II	13	65
III	1	5
Unknown	6	30
Mucin secretion		
Yes	0	0
No	11	55
Unknown	9	45

### Treatment

Treatment details and results are depicted in Table 2. All patients received standard RCT, consisting of an RT dose of 50.4-54.0 Gy and concurrent chemotherapy, most commonly capecitabine. All but one underwent surgery within 6–10 weeks (median: 8.7 weeks) after RCT completion, as per protocol. One patient was operated 21 weeks after RCT due to a femoral fracture and was excluded from the analysis. Ninety-five percent underwent curative (R0) resection and 80% had LAR.

**Table 2:** Treatment details and results

	**Number**	**%**
Radiotherapy dose (Gy)		
Median (range)	(50.4-54) 51.12	
Patients receiving 50.4 Gy	16	80
Chemotherapy		
Capecitabine	13	65
UFT	5	25
CI 5FU	2	10
Interval between RCT and surgery (weeks)		
Median (range)	8.7 (6.6 – 21.11)	
Surgery		
LAR	16	80
APR2	4	20
Surgical margins		
R0	19	95
R1	1	5
Pathological TNM stage		
0	4	20
I	5	25
II	3	15
III	7	35
IV	1	5
Tumor regression grade		
I	4	20
II	2	10
III	9	45
IV	5	25
V	0	0

Abbreviations: CI 5FU - continuous infusion of 5-fluorouracil.

UFT - tegafur-uracil, RCT – radiochemotherapy.

LAR – Low anterior resection, APR – Abdomino-perineal resection.

^1^One patient was removed from per – protocol analysis because of delayed operation.

^2^Including one patient who had APR and synchronous metastatectomy of liver metastasis.

**Figure 1:**
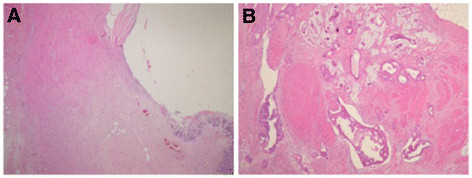
**The histopathological appearance of representative cases of a complete responder and a non-responder (H&E, magnification X4).** In the case of a complete responder (Figure 1**A**), the mucosa was eroded at the tumor site and there was marked fibrosis with focal microcalcifications. No residual tumor was found. In the case of a non-responder (Figure 1**B**), nearly no signs of response were noted (tumor regression grade [TRG] IV). Abundant tumor was found, with minimal fibrosis and regressive changes between the invasive tumor.

Following surgery, 4 patients (20%) had complete disappearance of their primary tumor (pT0) and 14 (70%) had no lymph node involvement (pN0). One patient had a single distant metastasis in the liver (pM1), which was found at surgery and was resected. Four patients (20%) achieved pCR and 6 (30%) had tumors with TRG I-II, grades that are considered as representing histopathological response. None had TRG V, i.e. all tumors had some response. The histopathological appearance of representative cases of a complete responder and a non-responder is presented in Figure 1.

### PET-CT evaluation

Data on individual patients’ metabolic and histopathological responses are shown in Table 3. The median SUV-MAX values at baseline and at the second scan were 20 (range, 7.5-56) and 17 (range, 5.8-37.9), respectively. Reduction of FDG uptake was observed in 14 patients (70%), with a maximum decrease of 45.2% (range: 15.3%-45.2%). In 5 patients (25%) SUV-MAX values increased, with a maximum increase of 85% (range: 1.6%-85.3%), and in one patient (5%) it remained unchanged. As mentioned, one patient was excluded from further analysis for delayed surgery.

**Table 3:** Individual patients’ metabolic and histopathological responses

**Patient Number**	**SUV-MAX baseline**	**SUV-MAX week 1**	**Absolute difference (ΔSUV-MAX)**	**Relative difference ΔSUV-MAX%))**	**TRG**	**Presence of pCR**
1	17.6	12	−5.6	−31.82	III	no
2 *	42	23	−19	−45.24	IV	no
3	21.3	19.4	−1.9	−8.92	I	yes
4	17	13	−4	−23.53	III	no
5	16.7	9.5	−7.2	−43.11	I	yes
6	17.8	13.5	−4.3	−24.16	III	no
7	13	18.5	5.5	42.31	II	no
8	15.2	15.2	0	0.00	II	no
9	20	13.4	−6.6	−33.00	IV	no
10	56	37.9	−18.1	−32.32	I	yes
11	23	26	3	13.04	III	no
12	28	36	8	28.57	III	no
13	18.2	15.4	−2.8	−15.38	III	no
14	16.9	13.7	−3.2	−18.93	III	no
15	11.9	9.7	−2.2	−18.49	IV	no
16	8.9	6.2	−2.7	−30.34	IV	no
17	7.5	13.9	6.4	85.33	IV	no
18	15.2	12.4	−2.8	−18.42	III	no
19	24.6	25	0.4	1.63	III	no
20	9.4	5.8	−3.6	−38.30	I	yes

Abbreviations: SUV-MAX - maximum standardized uptake value, pCR – pathological complete response.

*Patient was removed from per – protocol analysis because of delayed operation.

#### Correlation between metabolic and histopathological response

**Figure 2:**
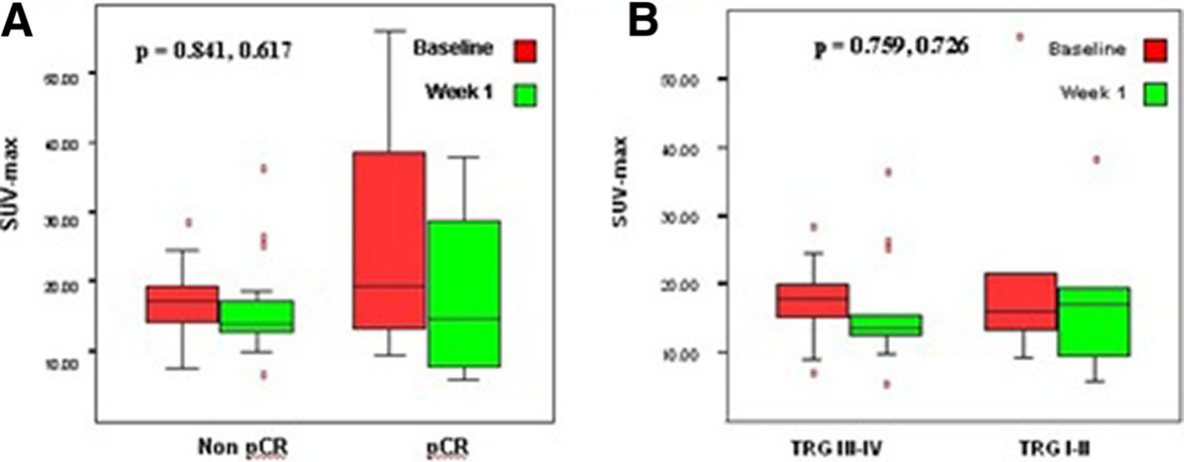
**Distribution of SUV-max values at baseline and after one week of RCT according to histopathological response.** Histopathological response was evaluated by the presence or absence of pCR (Figure 2**A**) or tumor regression grade (Figure 2**B**). Extreme values are represented by red circles.

The distribution of SUV-max values at baseline and after one week of RCT according to histopathological response is illustrated in Figure 2. There was no significant difference in SUV-MAX values at baseline or at day 8 (p = 0.617 and p = 0.841, respectively) between patients who did (n = 4) and those who did not achieve pCR (n = 15) (Figure 2A). Similarly, no correlation was found between SUV-MAX values at these time points and response per TRG. For example, patients with TRG I-II and those with TRG III-IV had almost identical values at both tests (p = 0.759 and p = 0.726, respectively) (Figure 2B).

**Figure 3:**
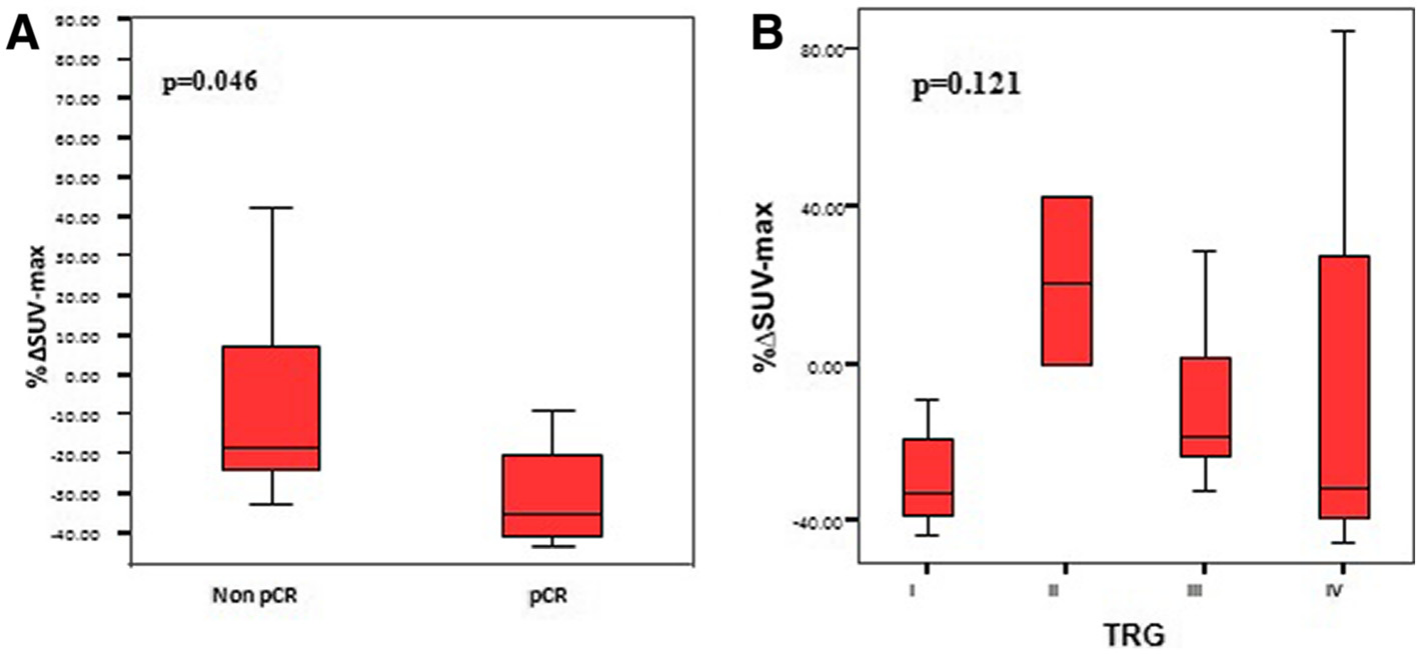
**Distribution of ΔSUV-MAX between baseline and after one week of RCT according to histopathological response.** Histopathological response was evaluated by the presence or absence of pCR (Figure 3**A**) or tumor regression grade (Figure 3**B**). Extreme values are represented by red circles.

We then evaluated the correlation between the extent of metabolic response after one week of RCT, i.e. ΔSUV-MAX values, and the histopathological response (Figure 3). We found a significant difference between patients who obtained pCR and those who did not: patients with pCR had a median ΔSUV-MAX of −35.3% (range: [−8.9%]–[−43.1%]) while patients without pCR had a median ΔSUV-MAX of −18.4% (range: [85.3%]–[−33%]) (p = 0.046) (Figure 3A). When comparing patients according to TRG, we noted a large variation between the different TRG categories (Figure 3B) and a similar decrease in SUV-MAX when comparing TRG I-II vs TRG III-IV or TRG I-III vs TRG IV (p = 0.483 and p = 0.841, respectively).

### Prediction of histological response

To find a cutoff value of ΔSUV-MAX that will predict pCR, ROC analysis was done. According to this analysis, any decrease in SUV-MAX between baseline and second PET-CT scans will predict all patients with pCR. Conversely, ΔSUV-MAX ≥0% will accurately predict failure to achieve pCR. The 0% cutoff provides a sensitivity of 100% and a specificity of 40% for not obtaining pCR, with a positive predictive value (PPV) of 31% and a negative predictive value (NPV) of 100%.

**Figure 4:**
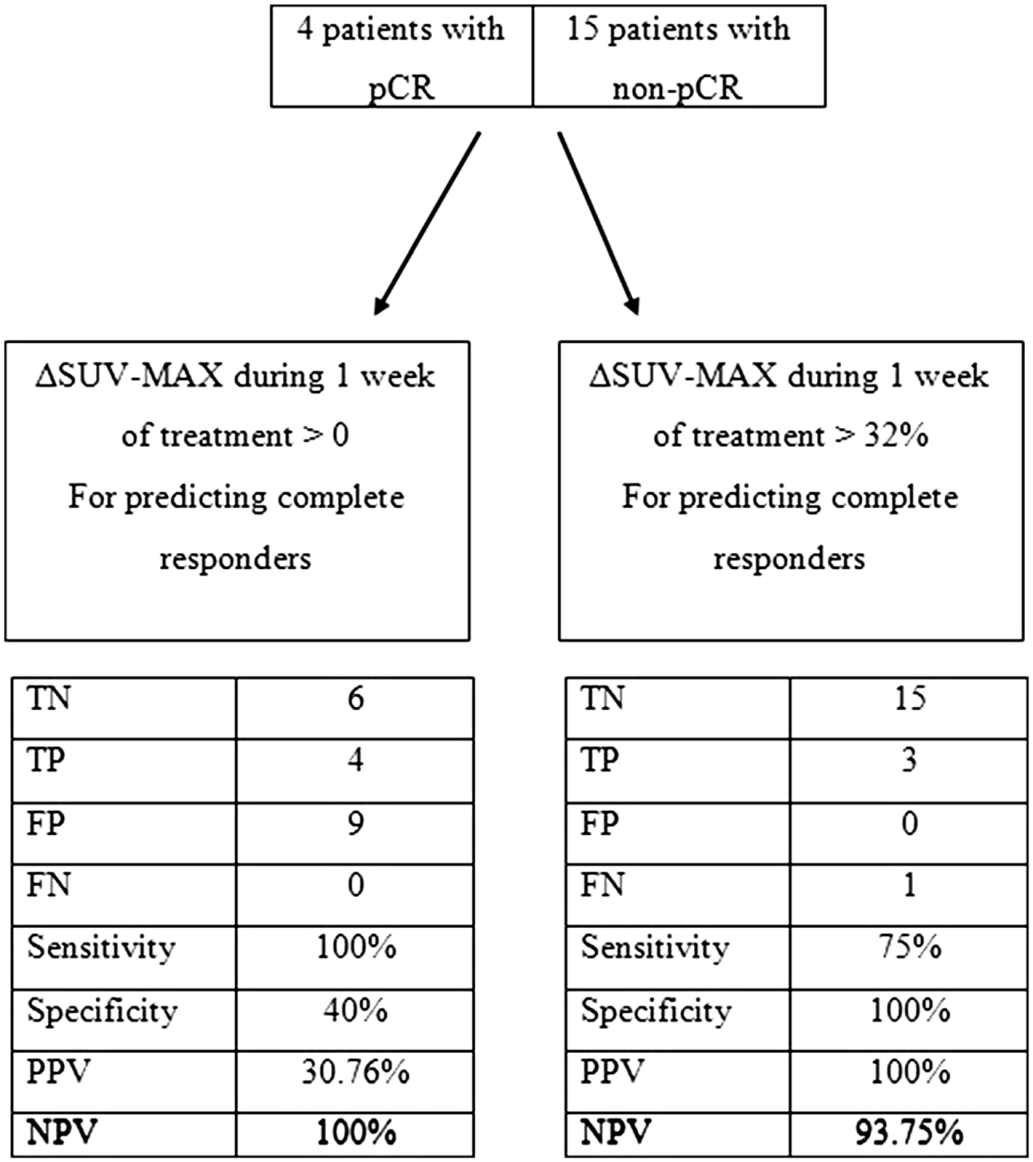
Prediction of histological response using early metabolic response.

On the other hand, a decrease of more than 32% will predict only patients with pCR. This cutoff gives a sensitivity of 75% and a specificity of 100% for achieving pCR, with PPV of 100% and NPV of 93%. The predictive value of metabolic response on pCR, using the two cutoffs, is summarized in Figure 4. A similar analysis was not done for TRG as no significant correlation was found between the SUV-MAX changes and that outcome.

## Discussion

Our results suggest that very early metabolic response, after one week of preoperative RCT for LARC, can indeed predict the achievement of pCR but not the TRG score. Furthermore, our findings are consistent with the accumulating evidence on the predictive role of repeating FDG-PET-CT during preoperative RCT for LARC. Seven other studies on this topic, providing data on 221 patients, were reported (Table 4). Five studies suggested a correlation between early metabolic response and histopathology at surgery and two were negative. The main differences between the studies were the timing of the second PET-CT, the definition of histopathological response and the cutoffs chosen. In most studies the subsequent PET-CT was done during or after the second week of RCT and in one study, by Janssen et al. [14], patients underwent two scans during RCT, after one and two weeks. There is no clear correlation between the results of the studies and the timing of the second PET-CT; yet, the only study in which PET-CT was repeated after three weeks was negative [16]. Data on a second PET-CT after one week of RCT are limited to two studies. Janssen et al. [14] reported some predictive impact of metabolic response after one week of RCT but due to overlap of ΔSUV-MAX values, no cutoff between responders and non-responders was identified. In our study, the differences in ΔSUV-MAX after one week of RCT showed good prediction of histopathological response. As the main benefit from early prediction of response is the ability to modify the treatment, one should attempt to predict this response as early as possible, even after one week. Our results, while still invalidated, support this approach.

**Table 4:** Correlation between metabolic and histopathological responses

**Author (country)**	**Year**	**N**	**2nd PET (weeks)**	**End-point**	**ΔSUV-MAX by response (%)**	**ΔSUV-MAX cut-off (%)**	**PPV (%)**	**NPV (%)**	**P value**
Cascini^13^ (Italy)	2006	33^1^	2	TRG	62 vs 28	52	100	87	<0.0001
Janssen^14^ (Netherlands)	2009	30	1	TRG^2^	29 vs 9	NA	NA	NA	0.013
			2	TRG	47 vs 18	43	91	82	0.001
Rosenberg^15^ (Germany)	2009	30	2	TRG	44 vs 30	35	82	58	0.085
Guerra^16^ (Italy)	2009	28	3	TRG	51 vs 43	49	75	42	NS
Lambrecht^17^ (Belgium)	2010	22^3^	2	pCR	59 vs 25	40	60	100	0.0036
Janssen^19^ (Netherlands)	2011	30	2	TRG	NR	48	100	79	<0.001
		21^4^	2	TRG	NR	48	83	93	0.001
Leibold^1^ (USA)	2011	27^5^	1.5	pCR	27 vs 23	NR	NR	NR	0.68
Current (Israel)	2011	20^6^	1	pCR	35 vs	32	100	94	0.046
				TRG	1832 vs 17	NR	NR	NR	0.064

Abbreviations: NR – not relevant, NA – not available.

^1^Patients received non-standard RCT, including also oxaliplatin and raltitrexed.

^2^A second end-point was prediction of ypT0-2 vs ypT3-4 by metabolic response.

^3^Two patients received non-standard RCT, including also oxaliplatin.

^4^The study included a validation cohort (n = 21); 5 patients were excluded from the analysis due to peritumoral inflammation.

^5^The study included 4 patients with metastatic disease; responders included complete and near complete pathological response (≥95% response).

^6^One patient was removed from per – protocol analysis because of delayed operation.

Another important dissimilarity among studies was the definition of histopathological response. Even the TRG classification varied: while four studies used the classical Mandard’s definition [21], one used the Becker’s definition [25]. Similarly, the three studies using pCR as endpoint were inconsistent: while we and Lambrecht et al. [17] defined response only as pCR, Leibold et al. [18] included also near pCR, i.e. extensive yet incomplete tumor destruction. It is unclear if the inclusion of tumors with less extensive and maybe slower response as “responding tumors” lead to the negative results of the latter trial. Obviously, each definition of histological response has advantages and disadvantages. For example, while predicting pCR may be more useful when one considers avoiding or minimizing surgery, it neglects patients with less extensive response who may still benefit from RCT. We believe that pCR is currently the adequate endpoint for future studies, as its prognostic impact is well established and its practical implications are clear [7,8,22,26,27].

The reported different cutoffs of 30-50% drop of SUV-MAX in the second PET-CT reflect multiple methodological variations between studies, including the PET-CT protocol and timing and the definition of histopathological response, as well as some intrinsic variability of the test. Most importantly, cutoffs were chosen from ROC curves, reflecting the investigator’s preference of the balance between the sensitivity and specificity of the test. Hence, the utility of two different cutoffs might be helpful. For example, if one is mostly concerned about false positive pCR, he may use a cutoff with maximal PPV (32% cutoff in our study); yet if false negative pCR is more critical, a cutoff providing maximal NPV (0% cutoff in our study) may be used. In any case, the positive studies reported relatively high PPV and NPV, and their cutoffs can be further optimized, considering the clinical needs, toward their validation.

Aside from their size, the most obvious limitation of the different studies, including ours, is the lack of validation. The first attempt to validate earlier results of the same group was reported recently [19]. In the first part of that study, enrolling 30 patients, the investigators determined the cutoff of ΔSUV-MAX after two weeks of RCT to be validated. The cutoff (48%) was indeed validated in an independent cohort of 21 patients, with high PPV (83%) and NPV (93%) (p = 0.001). Of note, 10% of the patients did not enter the analysis due to peritumoral inflammatory response [19]. Clearly, this study is a step in the right direction but more validation studies, on larger cohorts, are needed.

## Conclusions

In summary, this study shows the ability of a very early metabolic response, after only one week of preoperative RCT for LARC, to predict the achievement of pCR, but not TRG. If validated, it may enable practical modifications of the multi-modality treatment of LARC, such as referral of non-responders to undelayed surgery or avoidance of radical surgery in complete responders. A validation study is planned.

## Abbreviations

pCR: Pathological complete response; non-pCR: Non pathological complete response; ΔSUV-MAX: Δ - maximum standardized uptake value; TN: True negative; TP: True positive; FP: False – positive; FN: False – negative; PPN: Positive predictive value; NPV: Negative predictive value; TP/TP + FN: Sensitivity; TN/TN + FP: Specificity; TP/(TP + FP): Positive predictive value; TN/(TN + FN): Negative predictive value.

## Competing interests

The authors declare that they have no conflicting interests.

## Authors’ contributions

NG, YK, DG and BB designed the research. NG, OP, NG and BB analyzed the data. NG, YK, OP, HB, SM, NV and BB performed the research. NG, YK, OP, NG, and EI collected the data. NG, YK, AS, DG, and BB wrote the paper. All authors read and approved the final manuscript
